# Oxidative Stress and Atopic Dermatitis

**DOI:** 10.3390/antiox9030196

**Published:** 2020-02-26

**Authors:** Lucrezia Bertino, Fabrizio Guarneri, Serafinella Patrizia Cannavò, Marco Casciaro, Giovanni Pioggia, Sebastiano Gangemi

**Affiliations:** 1 Section of Dermatology, Department of Clinical and Experimental Medicine, University of Messina, 98125 Messina, Italy; bertino.lucrezia@gmail.com (L.B.); patrizia.cannavo@unime.it (S.P.C.); 2 School and Operative Unit of Allergy and Clinical Immunology, Department of Clinical and Experimental Medicine, University of Messina, 98125 Messina, Italy; mcasciaro@unime.it (M.C.); gangemis@unime.it (S.G.); 3 Institute for Biomedical Research and Innovation (IRIB), National Research Council of Italy (CNR), 98164 Messina, Italy; giovanni.pioggia@cnr.it

**Keywords:** atopic dermatitis, oxidative stress, advanced oxidation protein products, advanced glycation end products, superoxide, hydrogen peroxides, antioxidants, antioxidant enzymes

## Abstract

Atopic dermatitis is a common chronic/chronically relapsing inflammatory skin disease, with increasing worldwide prevalence. Etiopathogenesis is complex and multifactorial, with a mix of genetic, immunological and environmental aspects. Like in other chronic inflammatory diseases, oxidative stress plays an important pathogenetic role. We reviewed in vivo research studies on humans about oxidative stress and atopic dermatitis. Although sometimes contrasting, overall, they suggest that oxidative stress may have a significant role in atopic dermatitis, but our understanding is still incomplete, at least concerning in vivo data, because of limitations of available literature. Research consists of 33 papers published in 28 years, was not always performed on large study populations, represents a limited number of countries and ethnicities—not always in proportion to their size—and is scattered over multiple papers that, in the majority of cases, cannot be pooled and/or compared because many biomarkers were studied, in different tissues and with different methods. Further, larger studies appear warranted and necessary to shed more light on this aspect of atopic dermatitis, which is important not only to improve our understanding of this disease, but also for potential clinical and therapeutic implications.

## 1. Introduction

Atopic dermatitis (AD), also known as atopic eczema, is a chronic or chronically relapsing inflammatory skin disease, whose prevalence is continuously increasing. It affects all ages, from childhood (15–20%) to adulthood (1–3%). It is clinically characterized by skin dryness and itchy papules (occasionally vesicles in infants) that become excoriated and lichenified, often with crusting. Distribution is typically flexural, although it may vary, depending on age [1]. AD has a multidimensional burden, including a profound impact on the quality-of-life of patients and their families, a sometimes severe alteration of social functioning, and a significant amount of direct and indirect costs [2,3,4].

The pathogenesis of AD, not yet fully understood, is characterized by a complex interaction among genetic, immunological, and environmental aspects [5]. Genetic factors can be classified in two groups. The first group includes mutations of genes encoding for structural epidermal proteins or involved in the maintenance of epidermal barrier function. For example, it is known that 30–50% of AD patients have a loss-of-function mutation in the filaggrin gene (FLG); a mutation of the SPINK 5 (serine peptidase inhibitor Kazal-type 5) gene, encoding for antiprotease LEKTI (lympho-epithelial Kazal-type-related inhibitor), and associated with the initial phase of AD. The second group includes genes involved in the regulation of immune response, like interleukin (IL)-4, IL-4 receptor, IL-13, and the RANTES (Regulated on Activation, Normal T cell Expressed and Secreted) chemokine [6].

Additionally, the altered composition of intercorneocyte lipids also contributes to the disrupted barrier function in atopic subjects. This condition not only enhances transepidermal water loss, causing the typical skin dryness, but also leads to an increased penetration of allergens and pathogens, with consequent activation of the immune system [7]. Immune response of atopic subjects is dysfunctional, characterized by a Th2-dominated reaction and release of many proinflammatory cytokines, such as tumor necrosis factor (TNF) and interleukins (IL-4, IL-9, IL-22) [5,6]. While Th2 polarization is prevalent, particularly in the acute phase of the disease, significant Th17, Th22, and, in the chronic phase, Th1 responses can also be observed in patients. The type of immune polarization may vary depending on ethnicity and age [8,9].

Interaction between Th2 type inflammation and skin defects leads to chronicity of AD inflammation. Chronic skin inflammation is associated with overproduction of reactive oxygen species (ROS), such as superoxide (O_2_^−^) and hydrogen peroxide (H_2_O_2_). Exogenous factors, such as solar radiation or pollution, and/or psychological processes may also increase ROS concentration. Over time, accumulation of ROS eventually exceeds the defense capacity of the antioxidant system (AOS). This condition, defined oxidative stress (OS), plays a role in the pathogenesis of AD, as well as in other cutaneous and non-cutaneous diseases [10,11,12]. 

Atopic dermatitis is characterized by infiltration of inflammatory cells, such as lymphocytes, macrophages, eosinophils, and mast cells. In particular, macrophages have been observed to accumulate in large amounts in the acutely and chronically inflamed skin of patients with atopic dermatitis, playing a key role in the pathogenesis of this disease. They exert proinflammatory functions like antigen-presenting phagocytosis and the production of inflammatory cytokines and growth factors, so contributing to the characteristic chronic inflammation of atopic dermatitis [13]. In a murine model of AD, the chitinase 3-like 1 protein was shown to affect Th2 inflammation, M2 macrophage activation, and skin barrier function [14].

Components of the AOS include enzymes, such as superoxide dismutases (SOD), superoxide reductase, catalase (CAT), and peroxiredoxins and thioredoxin (TRX), as well as exogenous and endogenous nonenzymatic molecules, such as vitamins A, C, and E, uric acid, coenzyme Q10, and the whole glutathione system, which comprises glutathione (GSH) and the enzymes glutathione reductase, glutathione peroxidases (GPX), and glutathione S-transferases (GST) [15,16]. The direct impact of ROS in biological systems, i.e., modification of DNA, lipids, and proteins, can be evaluated through measurement of different biomarkers. Urinary 8-hydroxydeoxyguanosine (8-OHdG) is a biomarker of oxidative DNA damage and repair, whereas malondialdehyde (MDA), nitric oxide (NO), and 4-hydroxy-2-nonenal (HNE) are end products of lipid peroxidation, while advanced oxidation protein products (AOPPs) and advanced glycation end products (AGEs) derive from protein oxidation.

The purpose of this review is to summarize the studies available on the involvement of OS in AD, to better understand the pathogenesis of this disease, to identify useful OS biomarkers, and to discuss potential future treatment options.

## 2. Materials and Methods

The PubMed database (https://www.pubmed.gov) was used for this literature review. We searched for articles from inception to November 8, 2019, using the key terms of the MeSH (Medical Subject Headings) thesaurus related to AD and OS. The search string used was *(“atopic dermatitis” OR “eczema”) AND (“oxidative stress” OR “glycation end products, advanced” OR “malondialdehyde” OR “advanced oxidation protein products” OR “catalase” OR “myeloperoxidase” OR “glutathione peroxidase” OR “glutathione reductase” OR “glutathione transferase” OR “antioxidants” OR “superoxide” OR “hydrogen peroxides”)*.

We then read the abstract of each article whose title suggested that the association between AD and OS was analyzed. The entire article was read only if the abstract indicated that the article potentially met our inclusion criteria: English language, research papers, and studies on human population only. Finally, the references of the articles selected were examined in order to identify further studies that could be included in the review, based on the same criteria. 

Papers identified from the title, abstract, or full text as irrelevant to the topic in question, as well as those potentially relevant but performed on animals or cells, were excluded. 

## 3. Results

The initial PubMed search yielded 220 articles. Of these, 113 were not considered because the title and/or abstract suggested that they were not research papers, not written in English, not performed on human populations only, not relevant to the outcome of interest, and/or because the full text was not available. We then read the full text of the remaining 107 articles, and 74 were excluded: 60 because they were not relevant to the outcome of interest and 14 because they were not eligible for other reasons (studies performed on animals and/or cultured cell lines). 

For each of the 33 studies selected for inclusion in the review [17,18,19,20,21,22,23,24,25,26,27,28,29,30,31,32,33,34,35,36,37,38,39,40,41,42,43,44,45,46,47,48,49], Table 1 reports author(s) and year of publication, characteristics of the population studied (adults, children or both), number of AD patients and controls, type of sample analyzed to detect OS markers (blood, urine, skin biopsy, etc.), gene(s) studied (if any), OS markers examined and their concentration in AD patients and controls, any treatments or factors evaluated because of their possible influence on OS and/or AD, and the main outcome(s) of each study.

The articles considered span a 27-year period from 1992 to 2019. In detail, four studies were published in the 1990s, nine in the 2000s, and 21 in the last decade. Different geographical areas (and ethnicities) were not uniformly represented: 16 studies [17,18,23,24,27,32,33,34,35,36,37,38,40,42,45,46] were performed in Eastern Asia (8 in Japan, 6 in Korea, 2 in Taiwan), nine [21,28,31,39,41,43,44,47,48] in Europe (3 in Russia, 2 in Germany, 1 in Finland, Italy, Norway, Switzerland), four [20,22,29,30] in Middle Eastern countries (3 in Turkey, 1 in Israel), two [25,26] in Southern Asia (Bangladesh, India), and one [49] in the United States of America. Another study [19] included patients from multiple Canadian and European centers. Sample sizes were often small: ten papers [18,21,29,31,33,35,44,46,48,49] reported data concerning a population of less than 20 AD patients, eight papers [26,28,36,37,39,42,45,47] examined 20 to 40 patients, and only in seven cases [19,23,24,32,34,38,41,43] the number of patients was 100 or more. AD patients were adults (age >18 years) in seven studies, children in 19 (in four cases, patients were of neonatal age), both adults and children in eight. 

Studies were most frequently performed on blood samples (16 papers), skin biopsy or cutaneous parts (8 papers), and urine (6 papers). Cord blood was used in two cases, while placenta, cutaneous microdialysis, exhaled breath condensates, and ocular brush cytology were analyzed in one case each. Among the genes involved in the oxidoreductive balance, GSTM1, GSTT1, and GSTP1 were investigated, in five, four, and five papers, respectively. The number of biomarkers evaluated was overall rather large, but in the majority of cases each biomarker was evaluated in only one study; exceptions are MDA (assessed in six studies), 8-OHdG (five studies), GSH (four studies), alpha tocopherol, SOD and vitamin C (three studies), lipid peroxides, 4-HNE, ECP, MPO, total antioxidant capacity, vitamins A and E, Fe, and Zn (two studies). Assessment of the influence of pharmacological treatment or other factors (stress, environmental pollutants, experimental stimuli/conditions) on biomarkers was reported in four and six papers, respectively.

### 3.1. Genetics, Oxidative Stress, and Atopic Dermatitis

Seven articles included in our review analyzed the effects of interaction between different genotypes of GST and the predisposition to AD [17,19,32,36,38,41,43]. 

Vavilin et al. investigated genetic polymorphisms of GSTM1, GSTT1, and GSTP1 in Russian children. They found that only subjects with the GSTT1 null genotype (leading to lack of the corresponding enzyme) or the GSTM1 null-GSTT1 null-GSTP1 Ile105/Ile105 haplotype had a significantly higher risk of developing AD. Those with the GSTM1 active-T1 null-P1 Ile105/Ile105, GSTM1 null-T1 null-P1 Ile105/Val105, and GSTM1 null-T1 active-P1 Val105/Val105 haplotypes also showed a high risk, but the difference in comparison with healthy controls was only close to statistical significance [43]. Again, in Russian children, Safronova et al. evaluated the GSTP1 polymorphisms, showing a significantly lower risk of AD in subjects with the Ile105/Val105 haplotype, while the frequency of homozygotes Ile105/Ile105 and Val105/Val105 among AD patients was nearly significantly higher than among the controls [41].

Chung et al. investigated the effects of genetic polymorphisms of GSTM1, GSTT1, and GSTP1 on the risk of AD in Korean children of preschool age. AD patients had a much lower serum total antioxidant capacity than healthy controls, but no significant association was found between the disease and the genes studied (although possession of the Ile105Val haplotype, in homozygosis or heterozygosis, was very frequent among AD patients) [38]. Partially different results were reported by another group of Korean researchers, who found, in a population of children and adults numerically comparable to that examined by Chung et al., an association between GSTM1 null and AD, family history of AD, childhood onset AD, and AD without other allergic diseases, while the frequency of GSTT1 null was similar in patients and healthy controls [32].

Taiwanese studies included in our review were performed only on children: Wang et al. found that the GSTM1 null and GSTP1 Ile105/Ile105 genotypes were associated with an increased risk of AD, and maternal smoking habit during pregnancy influenced the genotype pattern susceptibility (cotinine levels of <0.1 ng/mL or >0.1 ng/mL were associated to higher risk of AD for subjects bearing GSTP1 Ile105/Ile105 or GSTM1 null, respectively) [36], while Wen et al. showed that children with a GSTM1 null-GSTT1 null genotype exposed in utero to perfluorooctanoic acid have a significantly increased risk for AD [17].

Finally, in a recent multicenter study on a large population of 5685 patients from Canada, Germany, Sweden, Netherlands, and Spain, a marginal genetic association was found between AD and the genetic risk score from nine single nucleotide polymorphisms within the GSTP1, TNF, TLR2, and TLR4 genes. In the same study, the authors also demonstrated a high susceptibility to traffic-related air pollution (evaluated from the quantity of NO_2_ at the home address of each child at birth) in atopic children with the TNF rs1800629 minor allele [19]. 

### 3.2. Biomarkers

In the papers considered in our review, MDA was the most frequently measured marker of oxidative stress, in different types of samples. Compared to those found in healthy controls, serum levels of MDA of AD patients were significantly higher in two of four studies [25,26] and not significantly different in the other two [22,38]. Urinary MDA was evaluated in one paper [37] and, although the difference between patients and controls was not significant, a correlation was observed between MDA levels and AD severity and extent. In exhaled breath condensates, the median levels of MDA were lower in AD patients than controls, with a difference close to statistical significance [28].

The second most frequently measured marker is urinary 8-OHdG. Levels of this marker were significantly increased in AD patients compared with healthy controls in three studies [42,45,46]. Conversely, two studies reported no significant differences between patients and controls [27,37], but one of them [27] demonstrated that subjects affected by AD have a significantly higher increase of 8-OHdG after exposure to environmental pollution (ultrafine particles, particle bound polycyclic aromatic hydrocarbons).

GSH was evaluated in serum in two papers, with contrasting results: Sivaranjani et al. [26] reported significantly lower quantities in AD patients compared with healthy controls, while Chung et al. [38] did not find differences between the two groups. Chang et al. [23] observed that placental levels of GSH were lowest among subjects who were exposed to prenatal maternal distress and later had AD.

Quantification of alpha tocopherol in AD patients also yielded variable results: Oh et al. [34] reported significant decrease of serum levels in comparison with healthy controls, Hoppu et al. [39], again in serum, found no significant difference, while Antille et al. [44] observed a significant increase of this biomarker in non-lesional skin samples of patients. Serum levels of vitamin C were assessed in three studies, of which two [25,26] reported significant decrease in AD patients, while the other one [34] did not show any significant difference from healthy controls. The three papers that showed a significant increase of 4-HNE in AD patients were performed in different types of samples, i.e., skin [40], ocular brush cytology [35], and exhaled breath condensates [28].

For some of the biomarkers evaluated in the two papers, results were univocal: AD patients had a significant increase of serum ECP [47,48], significant decrease of serum vitamins A and E [25,26] and Zn [25,30], as well as quantities of serum MPO comparable to healthy controls [47,48]. Conversely, partially variable or even opposite results were observed in studies on other biomarkers. Lipid peroxidation in skin samples was significantly increased in the population of AD patients examined by Sapuntsova et al. [31], but significantly decreased in the population examined by Antille et al. [44]. Decreased serum Fe levels in AD patients were reported by Amin et al. [25], while no difference vs. healthy controls was observed by Toyran et al. [30]. Serum SOD is significantly decreased in AD patients, according to the data published by Sivaranjani et al. [26]; however, according to Niwa et al. [40], the activity of the same enzyme in the skin is high in mild to severe AD but becomes significantly lower than in healthy controls in case of extremely severe AD.

Many other parameters related to oxidative stress were evaluated in only one of the papers corresponding to the selection criteria used for this review. In the serum of AD patients, significantly elevated levels of clusterin (correlated with disease severity) [24], significantly decreased levels of beta carotene [34], catalase [26], gamma tocopherol [39], glutathione peroxidase [26], Mg [30], NO [22], retinol [34], and levels of Cu similar to those of healthy controls [30] were found. Decreased also were, in AD patients, the serum total antioxidant capacity [38], serum disulphide, disulphide/native thiol ratio and disulphide/total thiol ratio [20], NO/melatonin ratio [22], nitrite/nitrate (NO_x_^–^) [45]. Polla et al. [49] found that peripheral blood mononuclear cells from patients with severe AD, but not peripheral blood neutrophils, when stimulated with phorbol myristate acetate/opsonized zymosan produce significantly more O_2_^–^ than their equally stimulated homologues from healthy controls.

Urinary levels of pentosidine [42] and biopyrrins [18] were higher in patients, while those of pyrraline [42] and selenium [45] did not differ significantly between patients and controls. In one study, urinary nitrate was found to be significantly higher in patients than in controls [37], while another study reported that the cumulative amount of urinary nitrite/nitrate (NO_x_^–^) was significantly lower in patients [45].

A study performed on cells from skin biopsies showed that basal generation of free radicals (such as superoxide anion radicals, hydroxyl radicals, peroxide radicals), peroxidation resistance of the substrate, and activity of antioxidant antiradical defense were significantly higher in AD patients than in controls [31]. Another study on skin biopsies revealed association between AD and elevated DNP, with direct correlation between the biomarker and disease severity [40].

Other data about AD patients were obtained with less common techniques: analysis of exhaled breath condensates, which showed an increase in 8-isoprostane, H_2_O_2_, and leukotriene B4 [28]; dermal microdialysis, which revealed an increase in isoprostanes and prostaglandins 9α,11α-PGF2α and PGE2 [21]; skin-wash sampling, which allowed to discover significantly reduced levels of uric acid, but total antioxidant scavenging capacity similar to that of healthy controls [29]; analysis of stratum corneum samples, which pointed out increased levels of SCCP in lesional skin compared to non-lesional skin or skin of healthy controls [33]; and ocular brush cytology, which demonstrated elevated quantities of hexanoyl-lysine [35].

### 3.3. Treatments of Atopic Dermatitis and Oxidative Stress

Many treatments are available for atopic dermatitis. However, researchers evaluated the effects on oxidative stress only for a few of them. In detail, only four of the papers included in this review reported the effects of various treatments on the biomarkers of oxidative stress studied and/or the risk of developing AD.

Tsukahara et al. [42] evaluated urinary pentosidine, pyrraline, and 8-OHdG in 12 children admitted to the hospital because of severe acute exacerbation of AD, with purulent skin infection caused by *Staphylococcus aureus*. In these children, pentosidine was significantly higher than in healthy controls and in patients with stable AD, while 8-OHdG was significantly higher than in controls. After seven to nine days of treatment with systemic antibiotics, topical antiseptics, and corticosteroids, a significant decrease of pentosidine and 8-OHdG was observed.

Oh et al. [34] studied the effects of the intake of vitamin E, beta carotene, folic acid, iron, and vitamin C in children. They found that high intakes of vitamin E, beta carotene, folic acid, and iron from diet and supplements were associated with a lower risk of AD, while the intake of vitamin C was irrelevant in this regard. Serum alpha-tocopherol and retinol were negatively associated with AD; the negative association between serum beta carotene and AD had marginal statistical significance, while vitamin C concentrations were similar in patients and controls.

Sapuntsova et al. [31], in a study on skin samples of adult patients, showed that treatment with thymodepressin i.m. significantly decreased lipid peroxidation, hydroxyl radicals, peroxide radicals, peroxidation resistance of the substrate, and activity of antioxidant antiradical defense, but not superoxide anion radicals. However, even after treatment, the levels of all of the above markers remained higher than those observed in healthy controls.

Quist et al. [21] investigated the effect of topical agents (tacrolimus 0.1% ointment or a lotion containing 12% ω-6 fatty acids) with potential to suppress AD inflammation. They assessed markers of OS (F2-isoprostanes, 5- and 8-prostaglandin F2α) and inflammation (9α,11α-prostaglandin F2α and prostaglandin E2) in the interstitial fluid collected in vivo via dermal microdialysis from lesional, non-lesional, and topically treated skin of AD patients. Levels of all eicosanoids were lower in non-lesional and tacrolimus-treated skin. Levels of total F2-isoprostanes, 9α,11α-prostaglandin F2α, and prostaglandin E2 were lower in non-lesional skin. Levels of 9α,11α-prostaglandin F2α were decreased in tacrolimus-treated skin compared with untreated AD skin.

## 4. Discussion

A critical analysis reveals that our understanding of the role of oxidative stress in atopic dermatitis is still incomplete, at least concerning in vivo data, because of several limitations of the available literature.

First, a higher number of researches would be expected, particularly when considering the magnitude and complexity of the topic: 33 publications between 1992 and 2019, or, in other terms, ~1.18 papers per year—on average—published worldwide, represent a rather small number, particularly when compared with the amount of studies on the role of oxidative stress in other diseases. A relatively important increase of the interest of researchers has been observed recently (20 papers published in the last ten years), and a further increase is desirable.

Second, the size of the study populations considered is frequently rather small. While this may still be adequate to demonstrate some associations (or lack of associations) between the disease and the biomarkers analyzed, larger studies would strengthen the conclusions drawn and might show other interesting elements, which become statistically detectable only in more numerous populations.

Third, the available studies were performed in a limited number of countries, and large areas and populations of the world are unrepresented in the literature. Moreover, the amount of papers published is sometimes not proportional to the geographical extension, number of inhabitants, or number of ethnicities of nations: as an example, the sum of AD patients from the United States of America [49] and India [26] ever studied concerning the association between oxidative stress and atopic dermatitis is 34, which is equal to the number of patients analyzed in a single Finnish paper [39] and only slightly superior to that (*n* = 33) of a single-center study performed in Italy [28]. Adequate representation of all ethnicities and areas is important in studies on the role of oxidative stress in diseases, because the oxidoreductive balance results from the complex interplay of three variables that may significantly change even within a small geographical area: genetics (with reference to the entire genetic structure of individuals, not only antioxidant enzymes), environment, and individual habits [50,51,52,53,54]. 

Last, but not less important, the many biomarkers of oxidative stress were studied in different tissues, with different methods, and with a different frequency, and were correlated with disease severity only in 3 of the 33 papers reviewed here. In the last years, some researchers reported how several tumors and carcinoma cell lines were characterized by elevated oxidative stress levels. The modified metabolism of aberrant cells involves mitochondria and oxygenation cycles provoking redox imbalance [55]. ROS and oxidative stress could favor genetic mutations strengthening malignant progression. However, serum biomarkers of oxidation (Advanced Glycation End Products and Advanced Oxidation Protein Products) demonstrated the importance of this stressing mechanism also on immune related diseases (i.e., autoimmune thyroiditis and chronic spontaneous urticaria). These pathologies, characterized by a chronic inflammatory activation, are a warning about how a loop made of damage and immune activation amplifies ROS effects on local and circulating products [56,57]. This situation represents well the complexity and multifaceted nature of the topic, and the consequent need to approach it from multiple points of view. However, on the other hand, these studies produced a multiplicity of data that, in the majority of cases, cannot be pooled and/or compared, thus leaving uncertainty about their generalizability.

Keeping these limitations in mind, some considerations on the relationship between oxidative stress and atopic dermatitis can still be made on the basis of available literature data. 

The role of polymorphisms of the GSTM1, GSTT1, and GSTP1 genes, the only ones investigated, appears unclear, because published studies report contrasting results about the association between the various genotypes and the disease. However, as already mentioned, maintaining the redox equilibrium is a multifactorial process of which GSTs are only a part, whose importance may vary depending on the interplay between all factors. It is well known that the GSTM1 null and the GSTT1 null genotypes are frequent in the general population, but with significant worldwide variability [58,59,60], and their role and “relative weight” as risk factors for several dermatologic conditions may range from irrelevant to highly significant, depending on the population studied [10,11,58,59,60,61,62,63,64,65]. Similar considerations may be applied to GSTP1 polymorphisms [66,67]. This further underlines the importance of the availability of data from different populations and areas. 

Sparse and sometimes contrasting were also the results of studies on biomarkers of oxidative stress, and, consequently, it is currently impossible to define a detailed profile of the alterations and their importance. However, when abstracting from specific cases, the overall picture is suggestive of a significant role of oxidative stress in the pathogenetic mechanism of atopic dermatitis: in the majority of papers, an increase in molecular signs of oxidation/oxidative damage and decrease in the antioxidant potential of the organism is reported. Indeed, this may be expected when considering that atopic dermatitis is a disease characterized by extensive chronic inflammation [50]. In agreement with this view, the few studies that evaluated the effects of AD treatments on oxidative stress reported that clinical improvement was accompanied by a reduction in the alterations of OS biomarkers, and that high intake of some antioxidants could decrease disease risk.

Other factors are also part of the complex interplay of oxidative stress [8] and would deserve better investigation in AD patients. Skin microbes can induce oxidative stress, and it is known that alterations of cutaneous microbial flora are associated with AD. Inflammation could be mediated, at least in part, by ROS released by monocytes activated by *Staphylococcus aureus*, which is notoriously increased on the skin of AD patients [68]. Vitamin D could play a potential role as an antioxidant factor, able to prevent membrane lipid peroxidation [69,70]. Notably, membrane lipid peroxidation was observed in AD patients [31], and a systematic review suggests that vitamin D supplementation may help ameliorate the severity of AD, although larger-scale and longer studies are necessary to confirm this conclusion [71]. Psychological stress in AD patients and in their mothers may also affect OS, but also this topic was scarcely studied [23,72]. 

In conclusion, further studies on a larger scale appear warranted and necessary to shed more light on the role of oxidative stress, a relatively overlooked aspect of atopic dermatitis. This is an open and promising field of research, difficult and fascinating at the same time, which will improve our understanding of the etiopathogenesis of this disease and has the potential to significantly change the clinical and therapeutic approaches, in the hopefully not too distant future.

## Figures and Tables

**Table 1:** Summary of the main data obtained from the studies included in the review.

Authors [reference]	Year	Study Population	No. of Patients	Tissue(s)	Gene(s)	Therapy or Other Factors	Markers of Oxidative Stress	Main Outcome (s)
Wen et al. [17]	2019	Children	45/839 AD	Cord blood	GSTT1, GSTM1	PFAS		In-utero PFOA exposure with GSST1/M1 null genotype is associated with AD
Shibama et al. [18]	2019	Adults	11 AD 7 HC	Urine, blood, skin			Urinary biopyrrins	Urinary biopyrrins correlate with serum IgE and TARC expression. In AD lesions, biopyrrins are strongly expressed and bilirubin oxidation augmented.
Huls et al. [19]	2018	Children	5685 *	Blood	GSTP1, TNF, TLR2, TLR4	NO_2_		Marginal association of a weighted genetic risk score from nine SNPs of the genes studied and its interaction with air pollution suggest the role of OS and inflammation in AD.
Uysal et al. [20]	2018	Children	60 AD 60 HC	Blood			Serum disulphide, disulphide/native thiol ratio, disulphide/total thiol ratio	OS and impaired dTDH are related to childhood AD. dTDH could be a possible diagnostic tool to predict AD chronicity.
Quist et al. [21]	2016	Adults	12 AD	Dermal microdialysis		Tacrolimus 0.1% ointment, 12% ω-6 fatty acid lotion	Isoprostanes, prostaglandins 9α,11α-PGF2α and PGE2	Eicosanoids are increased in lesional AD skin. Tacrolimus reduces OS and inflammation. No significant reduction of eicosanoids with ω-6 fatty acids.
Uysal et al. [22]	2016	Children	73 AD 67 HC	Blood			Melatonin, NO, MDA, NO/melatonin, MDA/melatonin	Serum melatonin levels higher in AD patients, but negatively correlated with disease severity. NO, NO/melatonin and MDA/melatonin lower in AD patients than in HC.
Chang et al. [23]	2016	Children	476 AD 2028 HC	Placenta, blood		Prenatal maternal distress	GSH, GSSG	11BHSD2 and GSH levels in the placenta were lowest among those who were exposed to prenatal maternal distress and later had AD.
Sol et al. [24]	2016	Children	100 AD 40 HC	Blood			Clusterin	Clusterin levels are higher in AD patients than in HC and correlate with disease severity.
Amin et al. [25]	2015	Children and adults	65 AD 65 HC	Blood			MDA, Vitamin A, E and C, Na, K, Ca, Zn, Fe	Higher levels of MDA and lower levels of vitamin A, E, C and Na, K, Ca, Zn, Fe in AD patients than in HC.
Sivaranjani et al. [26]	2013	Children and adults	25 AD 25 HC	Blood			MDA, SOD, CAT, GPX, GSH, Vitamin A, E, C	Increased MDA and decreased enzymatic and non-enzymatic antioxidants in AD patients.
Song et al. [27]	2013	Children	41 AD 43 HC	Urine		PMs, UFPs	8-OHdG	Increase of ambient UFPs causes within 24 hours an increase of urinary 8-OHdG in children with AD.
Peroni et al. [28]	2012	Children	33 AD 23 HC	Exhaled breath condensates			Leukotriene B4, 8-isoprostane, H_2_O_2_, MDA, 4-HNE	Significant decrease of pH and increase of leukotriene B4 and 8-isoprostane in AD patients. These could be markers of airway inflammation in children with AD.
Portugal-Cohen et al. [29]	2012	Adults	13 AD 17 HC	Skin wash sampling			Total antioxidant scavenging capacity, uric acid	TNFalpha, IL-1alpha, IL-6 and total scavenging capacity are not different between AD patients and HC. Uric acid is decreased in lesional skin and even more in non-lesional skin of AD patients compared to HC.
Toyran et al. [30]	2012	Children	92 AD 70 HC	Blood			Fe, Cu, Mg, Zn	Mg and Zn decreased in AD patients. No correlation of micronutrients with AD severity.
Sapuntsova et al. [31]	2011	Adults	18 AD 24 HC	Skin biopsy		Thymodepressin i.m.	Free radicals, LPO, intensity of generation of free radicals, concentration of lipid peroxides, peroxidation resistance of the substrate, activity of antioxidant antiradical defense	All parameters are significantly higher in AD patients before therapy than controls; after therapy, they all significantly decrease except the intensity of generation of superoxide anion radicals, but remain significantly higher than in controls.
Cho et al. [32]	2011	Children and adults	145 AD 267 HC	Blood	GSTM1, GSTT1			GSTM1 null genotype is associated with AD, family history of AD, childhood onset AD and AD without other allergic diseases. There is no correlation between AD and GSTT1.
Iwai et al. [33]	2010	Adults	17 AD 6 HC	Stratum corneum sample			SCCP	Increased SCCP in lesional areas of AD patients vs. non-lesional areas and healthy skin. Data suggest involvement of OS in the modification of stratum corneum protein and consequently in AD pathogenesis.
Oh et al. [34]	2010	Children	180 AD 242 HC	Blood		Vitamin E, beta-carotene, folic acid, iron, vitamin C	Alpha tocopherol, retinol, beta carotene, vitamin C	Intake of vitamin E, beta carotene, folic acid, iron is correlated with lower risk of AD. Alpha tocopherol and retinol concentrations are negatively associated with AD.
Wakamatsu et al. [35]	2010	Children and adults	14 AD 9 HC	Ocular brush cytology			HEL, 4-HNE	Lipid peroxidation and inflammation coexist in the conjunctiva of patient with AKC.
Wang et al. [36]	2010	Children	34 AD 106 HC	Cord blood	GSTM1, GSTP1	Prenatal smoke exposure	Cotinine	GSTM1 null and GSTP1 Ile/Ile genotypes are correlated with increased risk of AD. Cotinine levels influence the genotype pattern susceptibility.
Nakai et al. [37]	2009	Adults	21 AD 20 HC	Urine			Nitrate, MDA, 8-OHdG	Urinary nitrate is higher in AD patients than in HC. Nitrate and MDA levels correlate with AD severity and extent.
Chung et al. [38]	2009	Children	124 AD 260 HC	Blood	GSTP1, GSTT1, GSTM1		Total antioxidant capacity, MDA, GSH	Increased OS may play a role in AD pathogenesis. Total antioxidant capacity is lower in AD patients.
Hoppu et al. [39]	2005	Children	13/34 AD 34 HC	Blood			Alpha tocopherol, gamma tocopherol	Serum tocopherol levels are not associated with AD.
Niwa et al. [40]	2003	Adults	75 AD 15 HC	Skin biopsy			DNP, SOD, 4-HNE	Carbonyl moieties levels are directly correlated with AD severity. SOD activity is high in mild AD and progressively decreases with the increase of AD severity, becoming lower than that of HC in extra-severe AD.
Safronova et al. [41]	2003	Children	258 AD 157 HC	Blood	GSTP1			Both homozygotes GSTP1 (Ile 105 and Val 105) are at high risk of AD development (near significant differences), while the GSTP1-Ile105/Val105 genotype is significantly associated to resistance to AD.
Tsukahara et al. [42]	2003	Children	32 AD 30 HC	Urine		Systemic antibiotics, topical antiseptics and corticosteroids	Pentosidine, pyrraline, 8-OHdG	Pentosidine is higher during acute exacerbation of AD compared to stable AD and HC. 8-OHdG is increased in all phases of AD. Pentosidine levels are correlated with 8-OHdG and OS. In AD patients with acute exacerbation, pentosidine and 8-OHdG decrease after 7-9 days of treatment.
Vavilin et al. [43]	2003	Children	126 AD 199 HC	Blood	GSTM1, GSTT1, GSTP1			Significant association of AD with the GSTT1 null allele and the GSTM1 null-T1 null-P1 Ile/Ile haplotype, almost significant with the GSTM1 active-T1 null-P1 Ile/Ile, GSTM1 null-T1 null-P1 Ile/Val and GSTM1 null-T1 active-P1 Val/Val haplotypes.
Antille et al. [44]	2002	Children and adults	14 AD 14 HC	Skin scraping and biopsy			Alpha-tocopherol, lipid peroxides	In non lesional skin of AD patients, alpha-tocopherol is higher and lipid peroxides are lower than in HC, and there is an adaptive response to chronic inflammation.
Omata et al. [45]	2001	Children	27 AD 25 HC	Urine			8-OHdG, nitrite/nitrate, selenium	Impaired homeostasis of oxygen/nitrogen radicals and increased OS are involved in the pathophysiology of AD.
Tsuboi et al. [46]	1998	Children and adults	17 AD 17 HC	Urine, blood			8-OHdG	8-OHdG levels are an index of biochemical damage in AD and are positively correlated with log IgE.
Carlsen et al. [47]	1997	Children	34 AD 67 HC	Blood			s-MPO	ECP but not MPO reflect disease activity of AD.
Kristjansson et al. [48]	1994	Children	14 AD 15 HC	Blood			MPO	ECP is a good marker of ongoing asthma or AD.
Polla et al. [49]	1992	Children and adults	9 AD 9 HC	Blood		PMA OZ	O_2_^–^	PBMs from AD patients are primed for O_2_^–^ production and may participate, through this way, in AD pathogenesis.

* Total number of patients in the six pooled cohorts analyzed in the study. However, the type of data collected was not always the same in all cohorts. Symptoms of AD at age up to two years were found in 1146/5076 subjects, doctor diagnosed AD at age up to two years in 1090/5410 subjects, doctor diagnosed AD at 7 or 8 years of age in 440/5132 subjects. Abbreviations: AD = atopic dermatitis, AKC = atopic keratoconjunctivitis, C = controls, CAT = catalase, DNP = di-nitro-phenylhydrazone, dTDH = dynamic thiol-disulphide homeostasis, ECP = eosinophil cationic protein, GSH = glutathione, GPX = glutathione peroxidase, HEL = hexanoyl-lysine, HNE = hydroxy-2-nonenal, HC = healthy controls, LPO = lipid peroxidation, LDH = lactate dehydrogenase, MDA = malondialdehyde, MPO = myeloperoxidase, NO = nitric oxide, O_2_^−^ = superoxide, OS = oxidative stress, OXT = oxitocine, OZ = opsonized zymosan, PFAS = perfluoroalkyl substance, PFOA = perfluorooctanoic acid, PBMs = peripheral blood mononuclear cells, PMA = phorbol myristate acetate, SCCP = stratum corneum carbonylated protein, SOD = superoxide dismutase, TARC = thymus and activation regulated chemokine, 8-OHdG = 8-hydroxydeoxyguanosine, MDC = macrophage-derived chemokine, SNP = single nucleotide polymorphisms, 11BHSD2 = 11b-hydroxysteroid dehydrogenase type 2, PMs = particulate matters, UFPs = ultrafine particles, GSSG = glutathione disulfide.
